# The conformational dynamics of H2-H3n and S2-H6 in gating ligand entry into the buried binding cavity of vitamin D receptor

**DOI:** 10.1038/srep35937

**Published:** 2016-10-27

**Authors:** Wei-Ven Tee, Adiratna Mat Ripen, Saharuddin Bin Mohamad

**Affiliations:** 1 Institute of Biological Sciences, Faculty of Science, University of Malaya, 50603 Kuala Lumpur, Malaysia; 2 Allergy and Immunology Research Centre, Institute for Medical Research, Jalan Pahang, 50588 Kuala Lumpur, Malaysia; 3 Centre of Research for Computational Sciences and Informatics in Biology, Bioindustry, Environment, Agriculture and Healthcare (CRYSTAL), University of Malaya, 50603 Kuala Lumpur, Malaysia

## Abstract

Crystal structures of holo vitamin D receptor (VDR) revealed a canonical conformation in which the ligand is entrapped in a hydrophobic cavity buried in the ligand-binding domain (LBD). The mousetrap model postulates that helix 12 is positioned away from the domain to expose the interior cavity. However, the extended form of helix 12 is likely due to artifacts during crystallization. In this study, we set out to investigate conformational dynamics of apo VDR using molecular dynamics simulation on microsecond timescale. Here we show the neighboring backbones of helix 2-helix 3n and beta strand 2-helix 6 of LBD, instead of the helix 12, undergo large-scale motion, possibly gating the entrance of ligand to the ligand binding domain. Docking analysis to the simulated open structure of VDR with the estimated free energy of −37.0 kJ/mol, would emphasise the role of H2-H3n and S2-H6 in facilitating the entrance of calcitriol to the LBD of VDR.

The human vitamin D receptor (hVDR) is a ligand-dependent transcription factor that belongs to the nuclear receptor (NR) superfamily^1^. Similar to other members of NR superfamily, hVDR also shares a common modular architecture of a DNA-binding domain (DBD) linked to a moderately conserved ligand-binding domain (LBD)^1^,^2^ via a hinge region. LBD of NRs has a canonical structure of three-layer α-helical sandwich consists of about 13 α-helices and a three-stranded β-sheet. A hydrophobic ligand-binding cavity is located in the lower portion of the protein core without obvious entry site.

The first crystal structure of hVDR complexed with the active metabolite of vitamin D, calcitriol (1,25(OH)_2_D_3_) was solved in year 2000^3^. However, crystal structure of hVDR in apo state still remains elusive after 16 years. In other NRs, helix 12 (H12) of apo retinoid X receptor alpha (RXRα) is extended away from the LBD^4^,^5^ but folded against the LBD of holo retinoic acid receptor gamma (RARγ)^6^. These observations led to the mouse-trap model^6^ which proposes that large scale re-positioning of H12 seals the exposed binding cavity and stabilized by bound ligand to form a hydrophobic cleft known as activation function 2 (AF-2) for recruitment of coactivator^7^. Recently, it has been pointed out that the extended H12 observed in apo RXRα might be an artifact of crystal packing and extreme conditions during crystallization^8^,^9^. In most of the crystal structures available today, no significant structural differences can be distinguished between LBDs in the apo and holo states, particularly on H12 which packed against the LBDs^10^,^11^,^12^. As crystal structures represent only a transient conformation of biomolecules in their lowest states of energy, X-ray crystallography is limited in elucidating dynamic conformational changes.

Experiments that probe conformational dynamics of LBDs in solution such as nuclear magnetic resonance (NMR)^13^,^14^,^15^ and hydrogen/deuterium exchange (HDX)^16^,^17^ showed the lower portion of apo LBDs is more conformationally labile compared to the upper portion. Furthermore, agonist binding quenches dynamics of protein regions that form the binding cavity and H12. Despite these advances, we have little information on how dynamics of LBD facilitate ligand entry. In this work, we set out to investigate conformational dynamics of apo hVDR-LBD using multiple atomistic molecular dynamics (MD) simulations on the unprecedented timescale of microsecond. In addition, we used coarse-grained (CG) models of apo hVDR-LBD to accelerate sampling of conformational states. Our results suggest instead of H12 in the mouse-trap model, a dynamic region of hVDR-LBD is likely to allow ligand to enter the buried binding cavity.

## Results

### MD simulations reveal high fluctuation in H2-H3n and S2-H6 regions of apo hVDR-LBD

**Figure 1:**
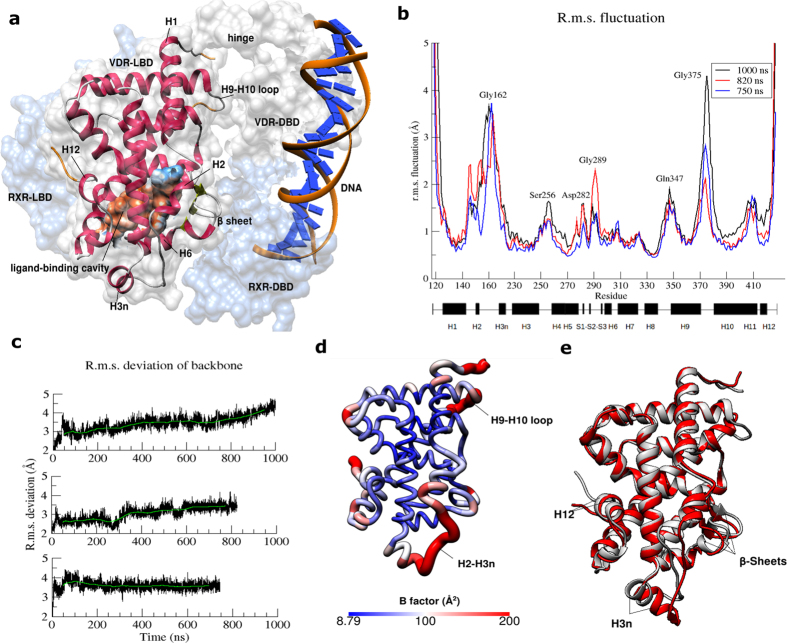
Atomistic MD simulations of apo hVDR-LBD. (**a**) Starting structure for MD simulations in ribbon and surface representation is shown together with other domains of hVDR, heterodimer partner RXRα and DNA to illustrate the full architecture of the complex. hVDR-LBD replaced LBD of peroxisome proliferator-activated receptors gamma (PPARγ) from a crystal structure of the complex^54^ (PDB: 3DZY). Unresolved amino acids in the LBD were built and colored in orange. Molecular surface of binding cavity is colored by hydrophobicity of amino acids according to the Kyte-Doolittle scale with colors ranging from orange red for the most hydrophobic to blue as the most hydrophilic. (**b**) R.m.s fluctuation (Å) of backbone atoms (N, Cα and C atoms) of each amino acid relative to starting coordinates. Secondary structures (H: α-helix; S: β-sheet) are indicated by black bars. (**c**) R.m.s. deviation of backbone of amino acids from starting coordinates. Running averages in green are calculated from 10000 frames. (**d**) B-factor value of amino acids (Å^2^) is mapped on the average structure. (**e**) Structural comparison of aligned average structure (red) and starting structure (grey).

We used the crystal structure of hVDR-LBD^3^ (PDB: 1DB1) as starting structure for MD simulations. The hollow ligand-binding cavity (Fig. 1a) has a volume of 953.7 Å^3^. The poorly structured VDR-specific insertion domain (Ser165-Pro215) which was excised prior to crystallography experiments is not included in this study. It was reported that removal of this insertion domain has no major effects on ligand binding and transactivation^3^.

We performed 3 independent atomistic MD simulations (1 μs, 820 ns and 750 ns) of apo hVDR-LBD with a total aggregate time of 2.7 μs and found the protein backbone between and inclusive of helix 2 and helix 3n (H2-H3n) and the loop between H9 and H10 (H9-H10 loop) exhibits high root mean square (r. m. s.) fluctuation (Fig. 1b). H2-H3n region is 29 amino acids of length from Tyr147 to Met226 with the highest fluctuation values correspond to three glycines (Gly162, Gly163 and Gly164). High fluctuation in H9-H10 loop (His371 to Leu378) from simulations is consistent with the unresolved amino acids in the crystal structure (Gly375 to His377). Except short helices H2, H3n, H11 and H12, helices are generally stable with r.m.s. fluctuation lower than 1.0 Å. Nevertheless, H11 and H12 are still relatively stable compared to the H2-H3n region and H9-H10 loop. R.m.s. deviation of protein backbone from initial structure was calculated separately for these atomistic simulations (Fig. 1c). Interestingly, r.m.s. deviation from initial structure in two longer atomistic MD simulations (1 μs and 820 ns) was not able to converge to a plateau. This is consistent with the view that apo LBD of NRs exhibits some properties of molten globule^8^,^18^, especially in the lower portion, probably due to an empty cavity in the hydrophobic core. This is to be expected as the protein domain in its initial holo conformation was simulated without the stabilizing ligand at the protein core.

Next, a representative model was built by averaging atomic coordinates from all sampled conformations throughout the simulation. B-factor value which measures the degree of atomic fluctuation in backbone of amino acids was mapped onto the average structure (Fig. 1d). Based on the model, helices forming the protein core and interior binding cavity are more stable than the exposed loops. Compared to the H2-H3n region, H9-H10 loop is too far away from the binding cavity to be directly implicated in ligand entry. However, H2-H3n region is unlikely to allow ligand entry just by itself based on visual inspection.

Multiple comparisons of the average structure to the initial conformation showed H2-H3n region tends to move away from the binding cavity in a robust manner (Fig. 1e, Supplementary Fig. S1). It was previously suggested that β sheet in hVDR-LBD is shifted outward to accommodate calcitriol^3^. In accordance to that, our results confirm that the β sheet moves inwards when ligand is absent. Only subtle changes can be observed in H12 in contrast to large conformational change by the extended form in apo RXRα^4^,^5^. Average r.m.s. deviation of H12 from starting coordinates was below 1.0 Å throughout the course of simulations (Supplementary Fig. S2).

**Figure 2:**
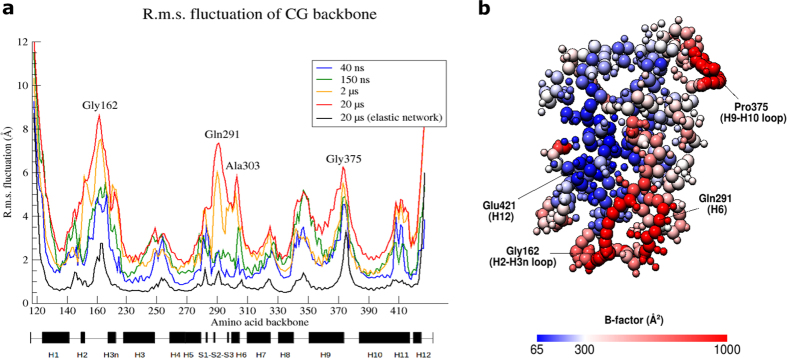
Apo hVDR-LBD in CG MD simulations. (**a**) R.m.s fluctuation (Å) of CG backbone of each amino acid relative to starting coordinates. For CG model, r.m.s. fluctuations from 3 simulations were averaged and indicated in red. The same analysis was performed and averaged from 19 simulations for CG + elastic model in black. (**b**) B-factor value of amino acids (Å^2^) from the 20 μs-long simulation is mapped on the average structure of CG apo hVDR-LBD. For each amino acid, a larger backbone bead and zero to four smaller side-chain bead(s) are depicted.

Using different initial coarse-grained structures of the protein domain, 25 CG MD simulations ranging from 40 ns to 20 μs were carried out. Due to the smoothened energy landscape in CG simulations, a time-scaling factor of 4 is typically used to approximate the corresponding time on atomistic timescale. We performed 3 CG MD simulations with each lasts for 20 μs, corresponding to a total of 240 μs of effective time sampled on atomistic timescale. Calculation of radius of gyration confirms the CG proteins were stably folded with the measure of compactness well within the narrow range of 18–19 Å, in agreement to a comprehensive analysis^19^ (Supplementary Fig. S3). Similar to atomistic MD simulations, r.m.s. deviation of CG protein backbone increased with time (Supplementary Fig. S4). Drastic rise of r.m.s. fluctuation in H2-H3n and S2-H6 backbones was observed only in CG MD simulations on microsecond timescale (Fig. 2a). The S2-H6 region consists of 18 amino acids (Trp286-Ala303) between and inclusive of the second β strand (S2) and helix 6 (H6). Differential dynamics in both regions might suggest the existence of slow protein motions that occur on timescale beyond nanoseconds. Mapping of B factor values from the 20 μs-long CG MD simulation indicates that the lower portion of the protein is more dynamic compared to the upper portion (Fig. 2b). Furthermore, we investigated the effects of applying additional elastic constraints onto the CG backbone (Supplementary Fig. S5) in 19 independent CG + elastic MD simulations of 20 μs long. The mean r.m.s. fluctuation of amino acid backbone from these CG + elastic MD simulations is significantly lower than that from atomistic and CG MD simulations (Fig. 2a). Nevertheless, most of the peaks and troughs were reproduced even when elastic constraints were imposed, crucially in the regions of dynamic H2-H3n and stable H12. In our subsequent studies, we focused on the dynamic properties of CG model based on two reasons – (i) the overall CG structure of apo hVDR-LBD remained well folded throughout multiple independent simulations and (ii) strong structural bias towards the initial CG model was introduced with elastic constraints, which is likely to cause this small globular protein domain to be overly rigid to investigate ligand access to the enclosed hydrophobic cavity.

### Principal component analysis (PCA) revealed backbone motion of H2-H3n and S2-H6

**Figure 3:**
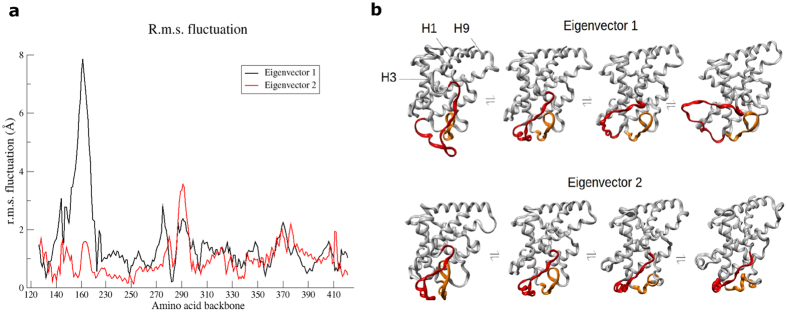
PCA of protein motions in 20 μs-long CG simulation. (**a**) R.m.s. fluctuation (Å) of protein backbone along motions in eigenvectors 1 and 2. Terminal loops (Asp118-Ser125 and Phe422-Ser427) were excluded from the analysis. (**b**) Projection of trajectory data on motion described by both eigenvectors. H2-H3n and S2-H6 are colored in red and orange respectively.

Instead of local harmonic fluctuations, we focused on the essential dynamics underlying the functional role played by the protein domain as a receptor. We performed PCA on trajectory data from a CG MD simulation of 20 μs long to determine the most dominant modes of motion (eigenvectors) of protein backbone. The first two eigenvectors out of 735 of them contribute to 38.4% of total positional fluctuation. As we have expected, H2-H3n and S2-H6 regions are implicated in eigenvector 1 and 2 with the highest r.m.s. fluctuation recorded at about 8.0 Å and 3.5 Å respectively (Fig. 3a). The trajectory was projected on both eigenvectors for visualization of the backbone motion (Fig. 3b). The rightmost structures of Fig. 3b show the extreme structures (extended structures) along the direction of collective motion described by eigenvector 1 and eigenvector 2. In the first eigenvector, the backbones of H1 and H2-H3n engage in a lever-like motion with H3 remains relatively fixed in position. As a result, the distance between H2-H3n and S2-H6 is varied. Compared to eigenvector 1, degree of backbone fluctuation in S2-H6 is only marginally higher in eigenvector 2, in which the backbone changes its position relative to H2-H3n. Visualization of both eigenvectors revealed backbone motions of H1 and H9 appeared to be coupled with those of H2-H3n and S2-H6. As H1 is located downstream of the flexible hinge which links LBD to DBD, it might be of interest to include protein motions from other domains of hVDR in MD simulations. It is important to note that eigenvector 1 and 2 only represent two of the most dominant degrees of freedom in the configurational space, so the projected structures do not necessarily correspond to physical structures sampled. Nevertheless, they highlighted the essential dynamics of H2-H3n and S2-H6 regions in hVDR-LBD.

### Molecular fluctuation in dynamic H2-H3n and S2-H6 forms flexible surface pockets

**Figure 4:**
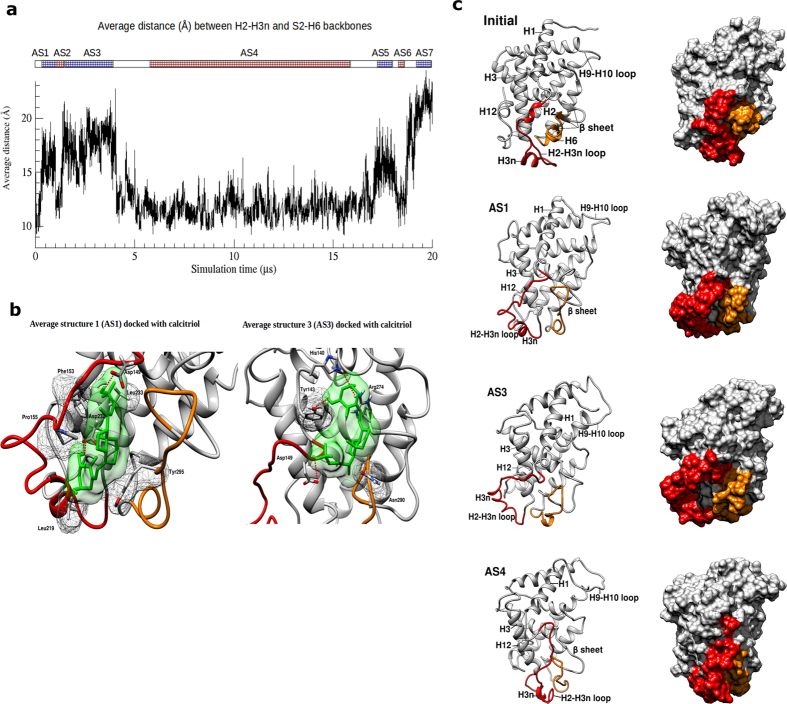
Dynamics in H2-H3n and S2-H6 create a flexible surface pocket. (**a**) Average distance (Å) between backbones of H2-H3n and S2-H6 changes with time. Color-coded bars (blue: opened state; red: closed state) indicate the time periods in which coordinates of all conformations are superimposed and averaged to produce a representative average structure (AS). AS1, AS3-AS5. (**b**) The initial structure (closed), AS1 (opened), AS3 (opened) and AS4 (closed) are shown in surface and ribbon representations with H2-H3n in red and S2-H6 in orange. (**c**) Molecular docking of calcitriol (green) to surface pocket of AS1 (above) and AS3 (below) is shown. Molecular surface of ligand is depicted as solid surface whereas the molecular surface of amino acids making hydrophobic interactions with the ligand is traced in mesh mode. Side-chains involved in forming hydrogen bond or hydrophobic interactions to the ligand are displayed in stick mode. Hydrogen bonds are indicated by red dashed lines.

We next sought to measure the average distance between the adjacent H2-H3n and S2-H6 regions against time in the 20 μs-long CG MD simulation. Distances between 15 pairs of amino acids (Supplementary Table S6) were calculated and averaged. Distance plot revealed both protein backbones underwent reversible conformational changes by moving closer (closed state) and further away (opened state) from each other (Fig. 4a). We obtained several representative CG average structures (AS1-AS7) in either state followed by transformation into atomistic representation using a published method^20^. Comparison between these average structures revealed a flexible surface pocket due to molecular fluctuation in the dynamic H2-H3n and S2-H6 backbones, close to the interior ligand-binding cavity (Fig. 4b). The surface pocket is evident in AS1, AS3 and AS5 when the average distance between H2-H3n and S2-H6 is at or above 15.0 Å. No surface pockets can be observed in AS2, AS4, AS6 and AS7 in closed state (Supplementary Fig. S7). Notably, none of these 7 average structures features a direct opening into the hydrophobic binding cavity of which the volume has shrunk drastically based on calculation (Supplementary Table S8).

Subsequently, molecular docking was performed to investigate the binding modes of calcitriol to surface pocket on AS1, AS3 and AS5. Docking analysis showed calcitriol not only docks to surface pocket on AS1 and AS3 with estimated Free energy of binding at −37.0 kJ/mol and −28.2 kJ/mol respectively, but also fits between the surface ridges created by H2-H3n and S2-H6. On the other hand, it binds to the outer side of H2-H3n ridge in AS5. For AS1, two hydrogen bonds were formed between the first and third hydroxyl groups with Asp232 and Asp149 respectively (Fig. 4c). Phe153 and Pro155 near the short and unwound H2 are involved in hydrophobic interaction with the side-chain and CD ring of the ligand. In another average structure with a different surface pocket, calcitriol is stabilized by four hydrogen bonds made with AS3 (Fig. 4c). Asp149 is again involved in hydrogen bond formation with the third hydroxyl group in the side-chain of calcitriol.

However, subsequent unbiased atomistic MD simulations of AS1 and AS3 with docked calcitriol were unable to result in the ligand reaching the interior cavity after 700 ns of simulation time. We reasoned that much longer simulation time in the realm of microsecond-to-milisecond timescale is required to reflect the binding kinetics of lipophilic ligands to the buried binding site. Moreover, interdomain interactions and protein motions transmitted from the hinge region to H2-H3n and S2-H6 via H1 might play an unknown role to facilitate ligand entry.

## Discussion and Conclusion

The use of vitamin D analogues for potential treatment of cancers^21^,^22^,^23^ and autoimmune diseases^24^,^25^ has been gaining momentum in preclinical research in addition to its classical treatment for osteoporosis. As supraphysiological doses of vitamin D cause hypercalcemia, potential analogues with reduced calcemic effects and improved efficacy are being actively developed. Despite this, in-depth knowledge on dynamics-function relationship of the receptor for vitamin D, particularly the understanding of ligand entry to the buried cavity at atomic level is still lacking. In this study, we utilized MD simulations to investigate the conformational dynamics of apo hVDR-LBD. Recognizing the importance of the yet unknown conformation of apo hVDR-LBD, we deprived the protein domain of stabilizing interactions from bound calcitriol to generate several representative atomistic and CG models converged on microsecond timescale for the first time. Based on calculation of r.m.s. deviation, both atomistic and CG models deviated significantly from their initial holo conformation when simulated without the ligand. H2-H3n was observed to be significantly more dynamic than the rest of the domain and moves away from the domain. Dynamic molecular fluctuation in H2-H3n from the simulation coincides with the unresolved connecting region between H1 and H3 in multiple crystal structures^26^,^27^,^28^ of its obligate heterodimer partner, RXRα (PDB: 1FBY; 1DKF; 1MVC), suggesting that this feature is not exclusive to hVDR. Furthermore, recent crystal structure of the intact multi-domain PPARγ^29^, liver X receptor (LXR)^30^ and hepatocyte nuclear factor 4-alpha (HNF-4α)^31^ with bound DNA (PDB: 3DZY; 4NQA; 4IQR) showed the region is not located in the domain interfaces but situated at the outward-facing tip of the LBD, away from the DNA. Hence, the dynamic H2-H3n of the protein domain is likely to be accessible for different ligands in solution.

As large-scale conformational changes that might allow ligand entry could not be fully explored in atomistic MD simulations, it is likely that the conformational changes occur on a much longer timescale. Also, ligand association and dissociation rates of nuclear hormone receptors are known to be several orders of magnitude slower than those from typical enzymes^32^. Due to the expensive computational cost posed by simulating an atomistic system on biological timescales, we used coarse-graining approach to remove the atomistic degrees of freedom for more effective sampling on the simplified energy landscape. From the CG MD simulations, we identified increased fluctuation in the backbone of H2-H3n and S2-H6 on the microsecond timescale. As both regions are adjacent to each other and in close proximity with the interior binding cavity, molecular fluctuation in these regions is likely to form a flexible docking station for a repertoire of natural and synthetic ligands. Throughout our CG MD simulations, the diminished hydrophobic binding cavity remained sheltered from solvated environment. This observation suggests ligand binds to the flexible surface pocket before inducing local conformational changes to wrestle into the protein core. Interestingly, results from docking simulations of calcitriol on the flexible surface pocket between H2-H3n and S2-H6 (Fig. 4c) are consistent with the alternative pocket found in hVDR more than a decade ago^33^. The alternative pocket which was suggested to be kinetically-favored by ligands mediating rapid nongenomic actions^34^,^35^,^36^,^37^ is situated between H2 and the beta sheet and partially overlaps the canonical binding cavity^33^.

Previously, it was reported that backbone motion of H12 of apo hVDR-LBD was not significantly different from the holo state during an atomistic MD simulation of 23 ns long^38^. Similarly, our result showed the extended conformation of H12 is not sampled in atomistic and CG simulations. In contrast, H12 was positioned closer to the empty binding cavity from our atomistic and CG MD simulations, compared to the initial structure. Furthermore, this observation is in agreement with a previous study on H12 of PPARγ which showed H12 is limited to local movements at ordinary temperatures^39^.

As summarized by several reviews^40^,^41^,^42^,^43^, none of the vitamin D analogues with promising result in preclinical research has been approved for clinical treatment of cancers. Limited information on the inherent dynamics of the apo receptor in binding event could be one of the factors that hinder development of steroidal and nonsteroidal analogues such as quercetin^44^. In this report, we propose that molecular fluctuation in the flexible H2-H3n and S2-H6 regions is likely to be instrumental for ligand access into the buried cavity, as opposed to large-scale conformational change of H12 in the mouse-trap mechanism^6^. We envisage that future *in silico* simulations of full-length receptor that include relevant motions from other domains and on longer simulation times will undoubtedly unveil a more accurate molecular mechanism underlying ligand entry into the apo hVDR-LBD.

## Methods

### Protein model for MD simulations

Crystal structure of apo hVDR-LBD with resolution at 1.80 Å was downloaded from Protein Data Bank (PDB) (PDB: 1DB1). Water molecules and calcitriol were removed from the crystal structure. Unresolved amino acids at both terminal loops (Asp118, Ser119, Asn424, Glu425, Ile426 and Ser427) and H9-H10 loop (Gly375, Ser376 and His377) were added to the structure using MODELLER^45^.

### Atomistic MD simulations

Atomistic MD simulations were carried out using GROMACS 5.0.4^46^ with CHARMM 22 forcefield^47^. The protein was centred in a periodic dodecahedron box and solvated with about 14,500 of water molecules. Ions were added to neutralized the system and resulted in approximately 47,800 atoms. Steepest descent energy minimization was carried out until the maximum force in the system is smaller than 1000 kJ mol^−1 ^nm^−1^. The potential energy of the energy-minimized system was approximately −8.00 × 10^5^ kJ/mol. Subsequently, equilibrations of the system were performed in the NVT ensemble at 300 K with V-rescale thermostat and NPT ensemble at 1 atm with isotropic Parrinello-Rahman barostat. Random velocities were assigned based on Maxwell-Boltzmann distribution to result in different initial configuration of systems. In each equilibration of 100 ps long, harmonic force constant at 1000 kJ mol^−1^ nm^−1^ was imposed on all non-hydrogen atoms for positional restraint. After equilibration, unrestrained production simulations were continued at the same temperature and pressure with a time step of 2 fs. All bonds were constrained using LINCS algorithm. For neighbor-searching algorithm, Verlet scheme was utilized and the cutoff distance for short-range electrostatic and van der Waals interactions was set at 10.0 Å. Long-range electrostatic interaction was computed using Particle Mesh Ewald method^48^.

### CG MD simulations and backmapping to atomistic structures

The structures of apo hVDR-LBD were extracted from atomistic simulation at an interval of 50 ns to construct a conformational ensemble. These atomistic structures were transformed to either CG or CG + elastic network models using MARTINI forcefield v2.2^49^. In both models, the CG protein was centred in a periodic dodecahedron box and solvated with 3000–4000 water beads and charge-neutralized by addition of counterions. Positional restrained energy minimization and equilibration were carried out at 300 K and 1 atm in 500 and 25,000 steps respectively. Group cutoff scheme was used for neighbor-searching with cutoff distance at 14.0 Å. Whereas cutoff distance for short-range shifted electrostatic and van der Waals interactions was set at 12.0 Å. Because a neutral CG water model was used, electrostatic interactions between charged beads were explicitly screened with a relative dielectric constant ε_r_ at 15. For production simulations, a time step of 20 fs was used due to reduced degrees of freedom in CG system. In CG + elastic network model, approximately 920 elastic constraints were generated globally for pairs of backbone beads (cutoff distance = 5 Å–9 Å) that are not already connected by bonded interactions. The force constant of elastic constraints was set to 500 kJ mol^−1 ^nm^−2^. After simulations, CG protein structures were backmapped to atomistic representations by geometric projection using a published method^20^, followed by energy minimization and short NVT equilibration.

### Post-simulation analysis

Calculation of r.m.s deviation and fluctuation, average structures and principal component analysis (PCA) were carried out using GROMACS. For PCA, trajectory data from CG MD simulation of 20 μs long was used. 245 backbone beads (Glu126 to Val421) excluding those in terminal loops were used to construct a 735 × 735 covariance matrix. Diagonalization of the covariance matrix with trace value of 32.6 nm^2^ yielded two principal components (eigenvectors 1 and 2) with eigenvalue of 9.36 nm^2^ (28.7% of total motility) and 3.16 nm^2^ (9.7% of total motility) respectively. Volume of surface pockets and interior cavities was measured using CASTp^50^ with a probe radius of 1.40 Å. VMD 1.9.2^51^ was used for visualization of simulation trajectory. Protein structures were visualized and figures were generated using Chimera 1.10.2^52^.

### Docking analysis

Flexible ligand-receptor docking was performed using AutoDock 4.2.6^53^ on average structures AS1, AS3 and AS5. The structure of calcitriol with 9 torsional degrees of freedom was obtained from the crystal structure of hVDR-LBD (PDB: 1DB1). Lamarckian Genetic Algorithm with a maximum of 2,500,000 energy evaluations was used for 200 runs. The grid box for docking analysis was centred at the lower portion of the protein encompassing H2-H3n and S2-H6 regions. Binding modes of calcitriol were ranked by estimated Free energy of binding.

## Additional Information

**How to cite this article**: Tee, W.-V. *et al.* The conformational dynamics of H2-H3n and S2-H6 in gating ligand entry into the buried binding cavity of vitamin D receptor. *Sci. Rep.* **6**, 35937; doi: 10.1038/srep35937 (2016).

**Publisher’s note:** Springer Nature remains neutral with regard to jurisdictional claims in published maps and institutional affiliations.

## Supplementary Material

Supplementary Information
