# Supplementary material for: The conformational dynamics of H2-H3n and S2-H6 in gating ligand entry into the buried binding cavity of vitamin D receptor
Source: Sci Rep. 2016 Oct 27;6:35937. doi: 10.1038/srep35937 (PMC5081507; doi:10.1038/srep35937)
Supplement: Supplementary Information [file srep35937-s1.pdf]

Title

The conformational dynamics of H2-H3n and S2-H6 in gating ligand entry into the buried binding cavity of vitamin D receptor.

Author list

Wei-Ven Tee[1], Adiratna Mat Ripen[2], Saharuddin Bin Mohamad[1,3]*

[1]Institute of Biological Sciences, Faculty of Science, University of Malaya, 50603 Kuala Lumpur, Malaysia

[2]Allergy and Immunology Research Centre, Institute for Medical Research, Jalan Pahang, 50588 Kuala Lumpur, Malaysia

[3]Centre of Research for Computational Sciences and Informatics in Biology, Bioindustry, Environment, Agriculture and Healthcare (CRYSTAL), University of Malaya, 50603 Kuala Lumpur, Malaysia

Correspondence should be addressed to Saharuddin Bin Mohamad; saharuddin@um.edu.my

a

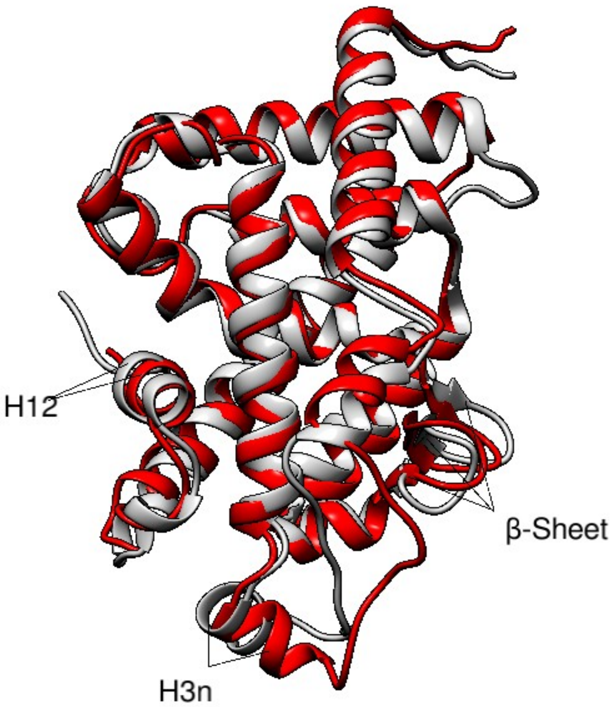

b

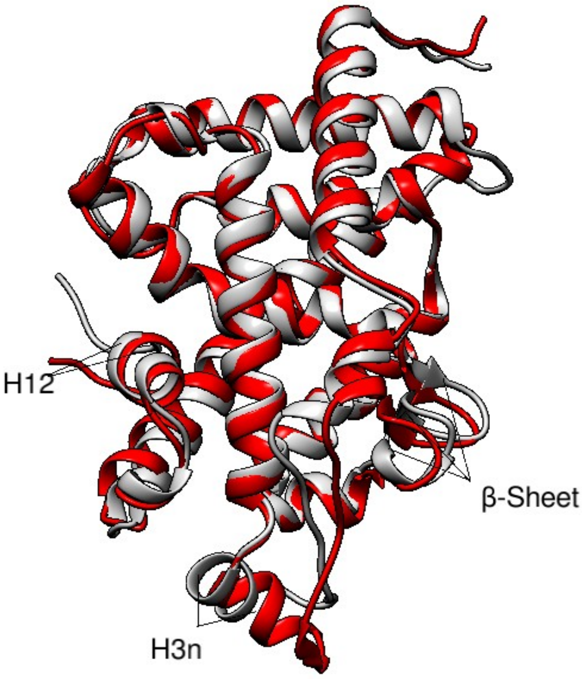

c

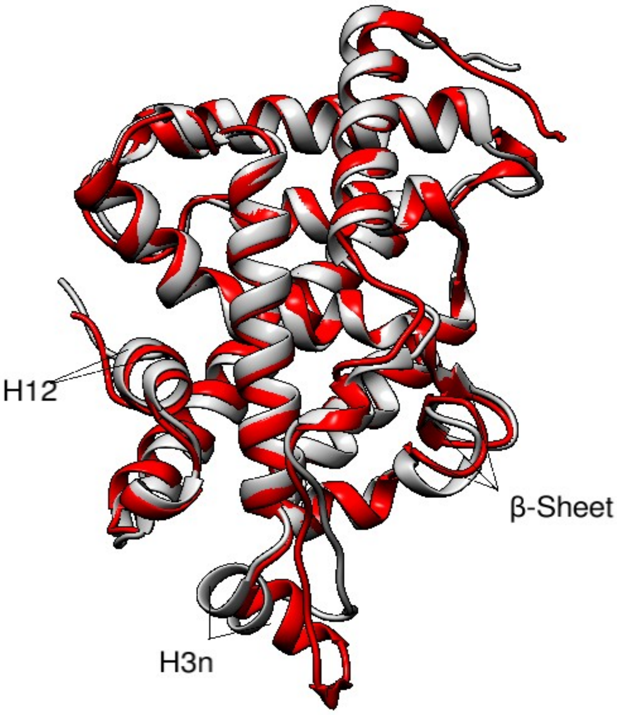

Supplementary Figure S1 | Structural comparisons between atomistic average structures and initial structure. a, Average structure from atomistic MD simulation of 1 μs in red is shown aligned with the initial structure in grey. **b** and **c**, **t**he same comparison using average structure from simulations of 820 ns and 750 ns long respectively.

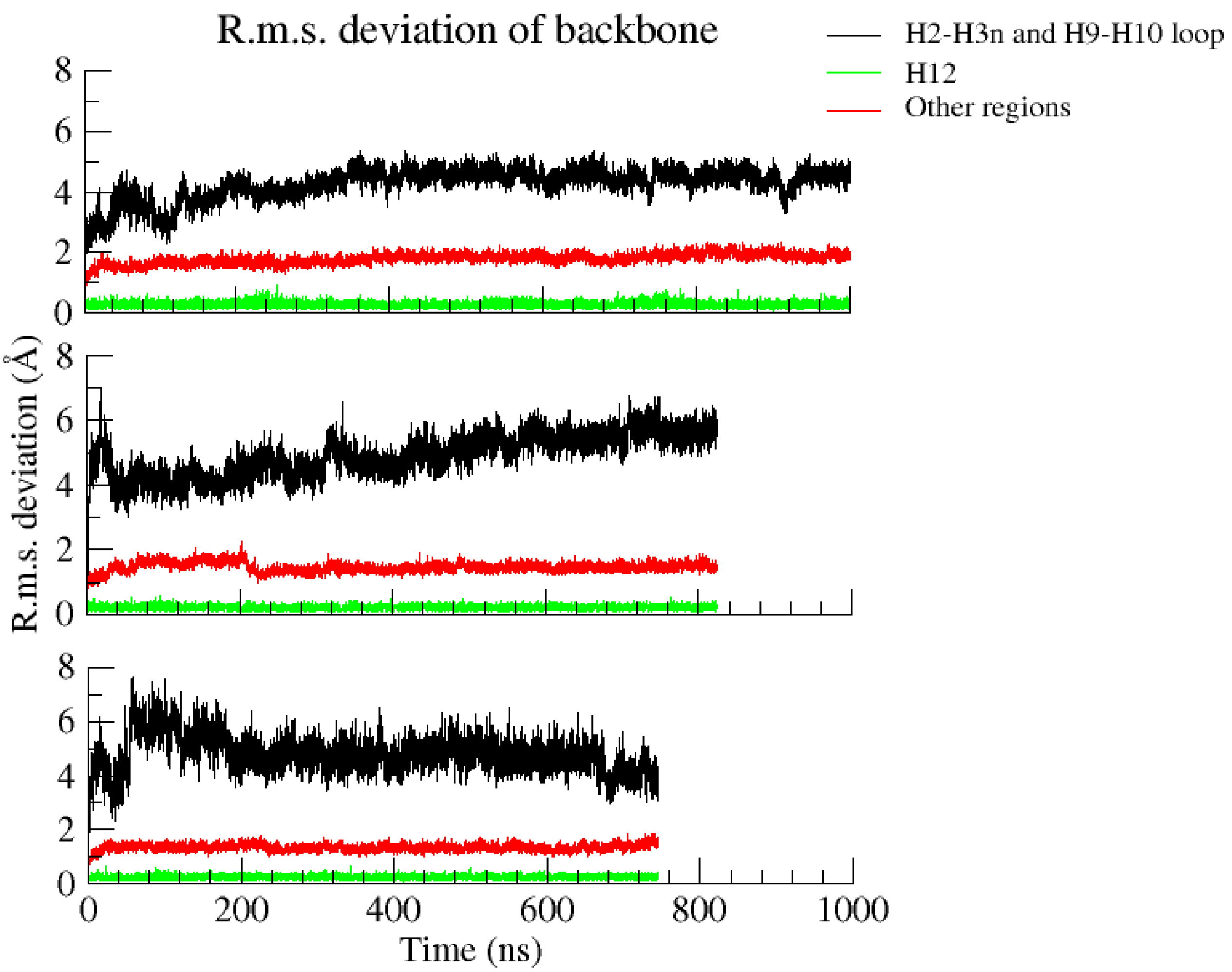

Supplementary Figure S2 | R.m.s. deviation of hVDR-LBD backbone in atomistic simulations. Only backbone atoms (N, Cα and C atoms) are included in the calculation. Amino acids in terminal loops (Asp118-Ser125 and Phe422-Ser427) were excluded from the analysis.

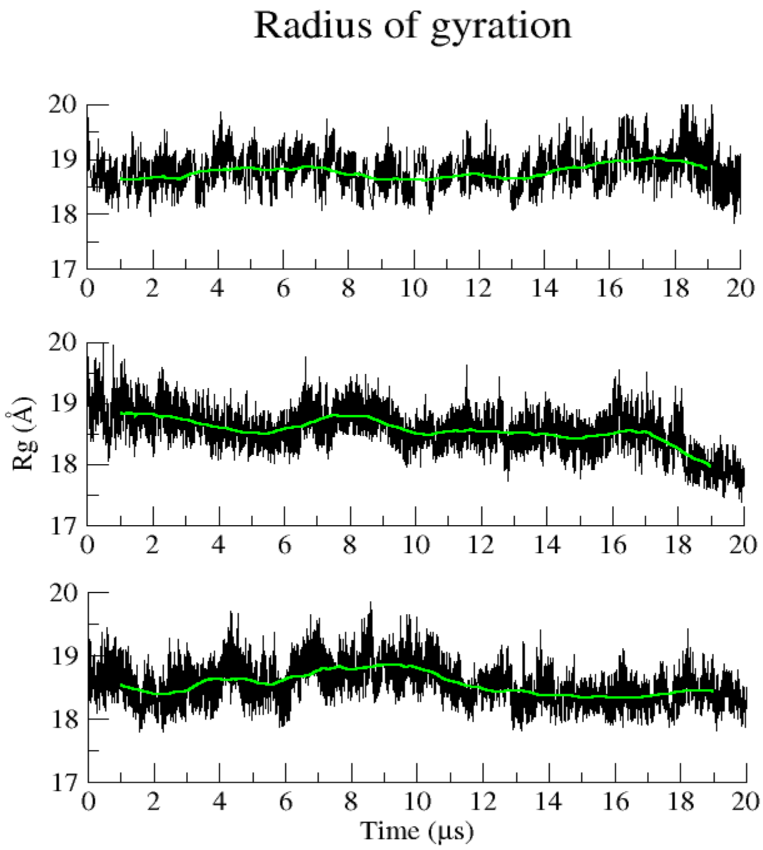

Supplementary Figure S3 | Radius of gyration analysis for CG MD simulations. Radius of gyration of CG apo hVDR-LBD plotted against time. Running averages colored in green are calculated from 100000 frames.

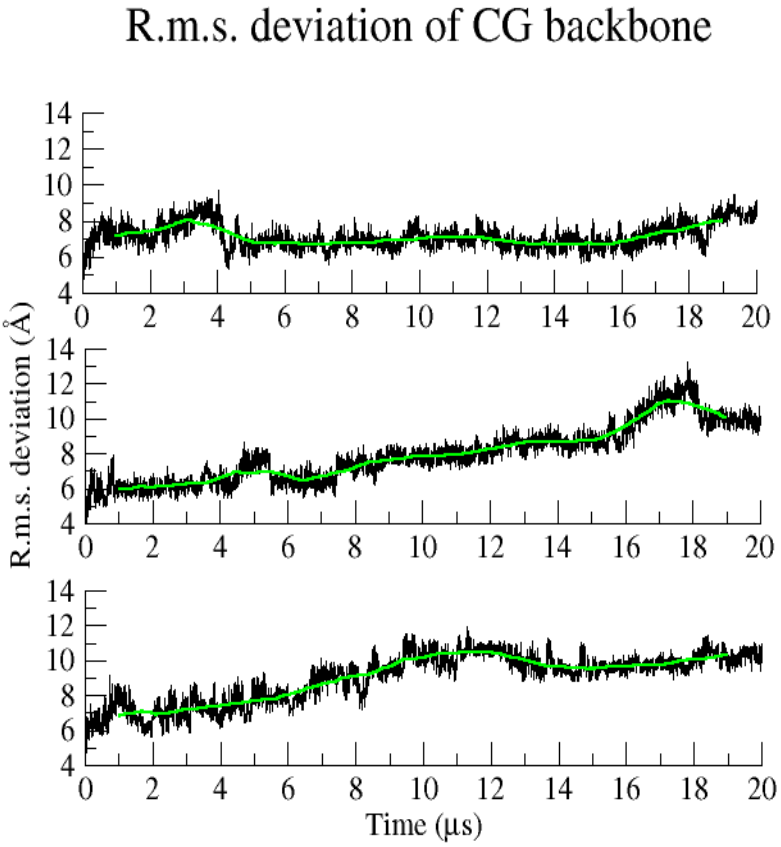

Supplementary Figure S4 | R.m.s. Deviation of CG backbone from initial holo conformation. R.m.s. deviation of CG backbone plotted against time. Running averages colored in green are calculated from 100000 frames.

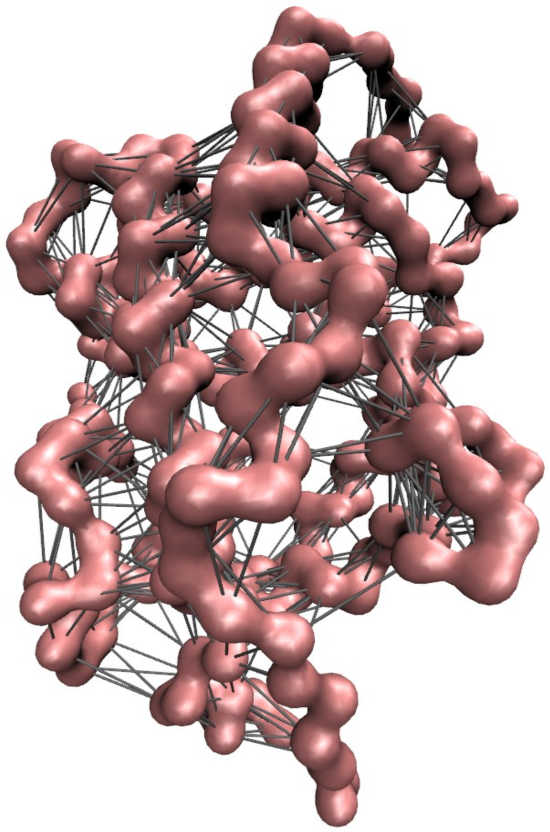

Supplementary Figure S5 | CG model of apo hVDR-LBD with elastic constraints. Only the CG backbone is shown together with elastic constraints depicted as black lines for amino acids backbone with cutoff distance of 5 - 9 Å .

**H2-H3n**	**S2-H6**
Tyr147	Trp286
Asp149	Thr287
Cys151	Cys288
Phe153	Gly289
Pro155	Asn290
Val157	Gln291
Val159	Asp292
Asp161	Tyr293
Gly163	Lys294
Ser216	Tyr295
Thr218	Arg296
Glu220	Val297
Ser222	Ser298
Leu224	Asp299
Met226	Ala303

Supplementary Table S6 | Backbone of amino acids selected for calculation of average distance between H2-H3n and S2-H6 in 20 μs-long CG MD simulation.

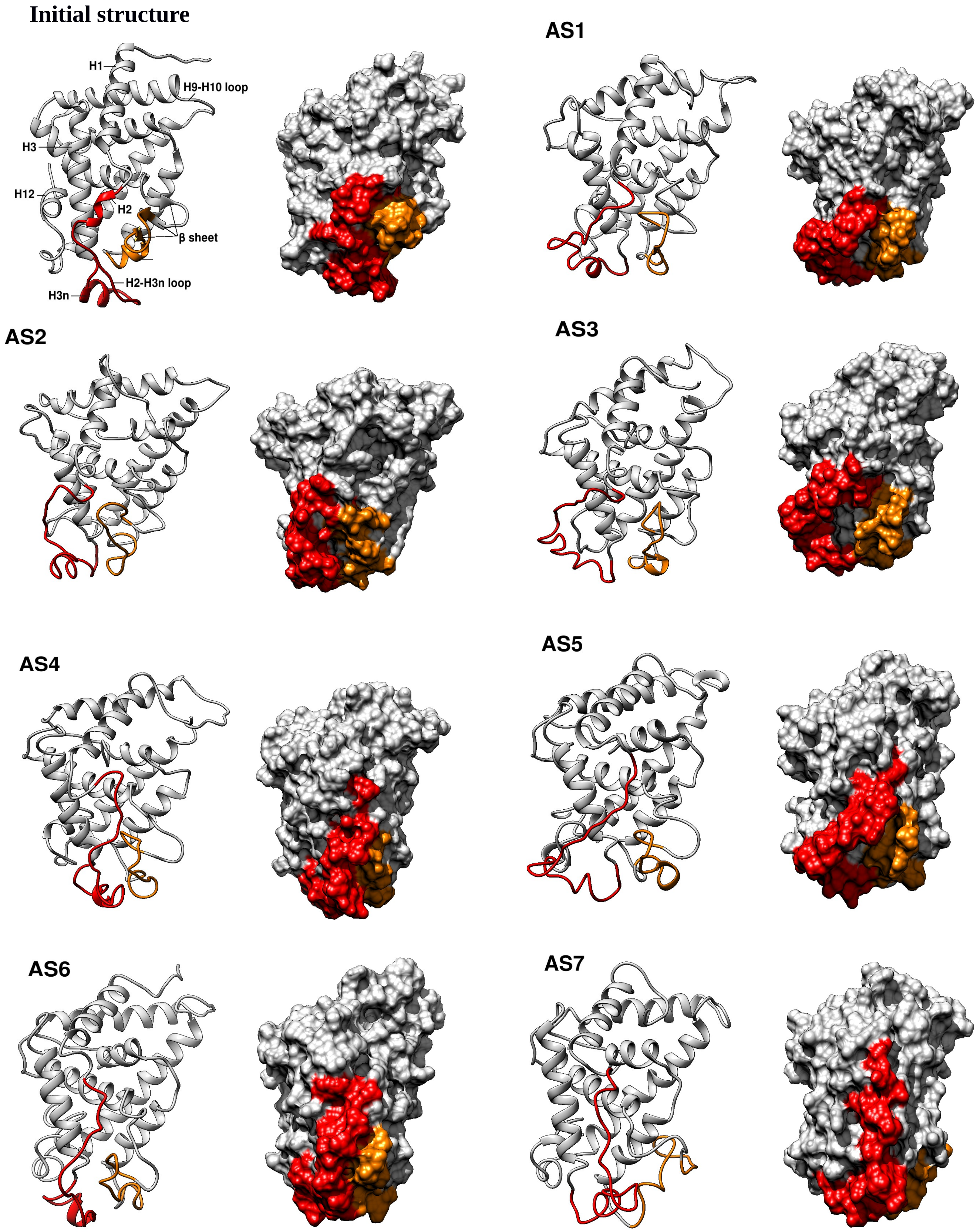

Initial structure
H1
H9-H10 loop
H3
H12
H2
β sheet
H2-H3n loop
H3n
AS1
AS2
AS3
AS4
AS5
AS6
AS7

Supplementary Figure S7 | Average structures in ribbon and surface representation. The initial and average structures (AS1-AS7) are shown in ribbon and surface representation. AS1, AS3, AS5 represent the open form of apo hVDR-LBD whereas AS2, AS4, AS6 and AS7 indicate the close form. Surface pocket is formed at the lower portion of the domain when H2-H3n (red) moves away from S2-H6 (orange) in the open form.

	**Area (solvent accessible) (Å^2)**	**Area (molecular surface) (Å^2)**	**Volume (solvent accessible) (Å^3)**	**Volume (molecular surface) (Å^3)**	**No. of openings to external surface**	**Volume (molecular surface) of interior ligand-binding cavity (Å^3)**
AS1	111.6	259.1	63.0	310.5	1	419.2
AS2	52.8	134.6	24.4	146.1	2	189.5
AS3	149.9	288.0	72.6	375.8	1	258.9
AS4	14.1	57.5	4.3	50.0	1	153.3
AS5	186.6	411.8	69.2	465.7	3	188.5
AS6	9.3	87.8	1.0	61.4	0	209.1
AS7	13.1	87.0	2.3	61.4	0	203.0

Supplementary Table S8 | Area and volume of the buried ligand-binding cavity and surface pocket or cavity formed between H2-H3n and S2-H6.
